# Inhibition of Lipolysis by Mercaptoacetate and Etomoxir Specifically Sensitize Drug-Resistant Lung Adenocarcinoma Cell to Paclitaxel

**DOI:** 10.1371/journal.pone.0074623

**Published:** 2013-09-11

**Authors:** Jiajin Li, Shiyan Zhao, Xiang Zhou, Teng Zhang, Li Zhao, Ping Miao, Shaoli Song, Xiaoguang Sun, Jianjun Liu, Xiaoping Zhao, Gang Huang

**Affiliations:** 1 Department of Nuclear Medicine, Ren Ji Hospital, School of Medicine, Shanghai Jiao Tong University, Shanghai, China; 2 Institute of Health Sciences, Shanghai Jiao Tong University School of Medicine & Shanghai Institutes for Biological Sciences, Chinese Academy of Sciences, Shanghai, China; Univesity of Texas Southwestern Medical Center at Dallas, United States of America

## Abstract

Chemoresistance is a major cause of treatment failure in patients with lung cancer. Although the extensive efforts have been made in overcoming chemoresistance, the underlying mechanisms are still elusive. Cancer cells reprogram cellular metabolism to satisfy the demands of malignant phenotype. To reveal roles of cancer metabolism in regulating chemoresistance, we profiled the metabolic characteristics in paclitaxel-resistant lung cancer cells by flux assay. Glucose and oleate metabolism were significantly different between resistant and non-resistant cells. In addition, targeting metabolism as a strategy to overcome drug resistance was investigated using specific metabolic inhibitors. Inhibition of glycolysis and oxidative phosphorylation by 2-deoxyglucose and malonate, respectively, potentiated the effects of paclitaxel on nonresistant lung adenocarcinoma cells but not paclitaxel-resistant cells. By contrast, inhibition of lipolysis by mercaptoacetate or etomoxir synergistically inhibited drug-resistant lung adenocarcinoma cell proliferation. We conclude that lipolysis inhibition potentially be a therapeutic strategy to overcome drug resistance in lung cancer.

## Introduction

Lung cancer is world widely the leading cause of cancer-related death. Because of the lack of symptoms at an early stage, the majority of newly diagnosed patients have locally advanced or metastatic tumor, and require systemic treatment. Therefore chemotherapy is the major treatment of lung cancer. However, the prognosis of lung cancer is still poor. The median survival time of about 18 months in inoperable stages [1]. Acquired or inherent drug resistance of cancer cells is a major cause of failure in chemotherapy. The ability to reduce chemoresistance would be of great benefit to cancer patient.

Cancer cell biology and phenotypic traits are greatly influenced by the changes in energy metabolism. Mounting evidence supports the idea the unique metabolic profile of cancer is linked to drug resistance in cancer therapy [2]. It has been shown that efficient cellular scavenging of chemo drugs induced reactive oxygen species (ROS) at least in part contribute to drug resistance. And the mechanism may be that in chemo-resistant cells, electron leakage from respiratory chain complexes and thus the formation of ROS by electron transport chain (ETC) is interrupted [3]. Recent evidence suggests that targeting the cancer-specific metabolic pathway may offer selectivity in cancer treatment [4].

Drug resistant tumor cells display increased dependence on fatty acid oxidation (FAO) and glycolysis, which likely compensate for the reduction in cellular ATP production and produce intermediates to support cellular growth [5,6]. This metabolic shift releases drug resistant cells from the typical restraints, and provides a potential way for treatments. It was reported that carnitine palmitoyltransferase 1C (CPT 1C) overexpression in cancer is important for cancer cell survival and resistance to therapy [7]. In addition, compounds that target dysregulated cellular metabolism often have the ability to influence the effect of current anticancer treatments [2]. Several mechanisms contribute to chemo resistance, such as alteration in drug transport and metabolism, mutation and amplification of drug targets, as well as defects in functional pathways having a key role in cell growth arrest or death and DNA repair [8,9]. Yet it remains an open question whether the dysregulated cellular metabolism contributes to therapeutic resistance or only is a subsequent phenomenon of resistance.

The lessons we have learned in the past, therapeutic strategy based on single target, such as a metabolic enzyme or a signal transducer hardly cures cancer. The combination of metabolic inhibitors and chemo drugs may become a promising solution for chemoresistance [10]. This study was conducted to gain insight into which type(s) of metabolic inhibitors could reverse resistance of lung adenocarcinoma cell to paclitaxel, a widely used chemotherapeutic drug for lung adenocarcinoma. We determined the effects on cell proliferation by inhibitors of glycolysis, oxidative phosphorylation and fatty acid oxidation combined with paclitaxel in drug-resistant lung adenocarcinoma cell A549/Taxol and the parental A549 cell line.

## Materials and Methods

### Materials

Cell culture reagents (DMEM and fetal bovine serum) were from Invitrogen/Gibco. [1-^14^C] oleate (OA) and [1-^14^C]-glucose were from Shenzhen Zhonghe Headway Bio-Sci & Tech Co. 2 –deoxyglucose(2DG), malonate (Malo), mercaptoacetate (MA) and etomoxir were obtained from Sigma-Aldrich, and paclitaxel (PTX) was from Bristol-Myers Squibb.

### Cell culture

Paclitaxel-resistant A549T and parental non-resistant A549 lung adenocarcinoma cell lines [11] were kindly gifted from Institute of Thoracic Tumor (Shanghai Chest Hospital, Shanghai, China). The cells were incubated in DMEM medium. The media were supplemented with 10% FBS and 100 units/mL penicillin/streptomycin. Cell cultures were maintained in 5% CO_2_ and air in a humidified 37°C incubator. Cells plated in plastic culture dishes were treated with drugs 1 day after plating, and the drugs were present throughout the indicated incubation periods.

### Glucose and Oleate oxidation

The incorporation of [1-^14^C] oleate or [6-^14^C] glucose into ^14^CO_2_ was determined as previously reported [12]. Briefly, cells were cultured in 10 cm^2^ dishes, and the cells were exposed to DMEM supplemented with [1-^14^C] OA (0.1 µCi/ml) or [6-^14^C] glucose(0.1 µCi/ml). The dish was placed in a container to collect CO_2_ produced. Rates of oleate or glucose consumption were measured by incubating cells for 120 min at 37°C. Fresh air was pumped into the container by a ventilator. The [^14^C]CO_2_ was driven into a vial and trapped by Hyamine hydroxide. No-cell controls were included to correct for unspecific CO_2_ trapping.

### Cell Fractionation and Separation

Mitochondria and nucleus were rapidly isolated by differential centrifugation essentially as described [13,14]. Briefly, cells were washed twice with ice cold PBS, resuspended in PBS, broke open cells to release nuclei using a pre-chilled homogenizer. Samples for protein assay were taken and the homogenized cells were then centrifuged at 1000 g for 10 min at 4°C to pellet nuclei and other fragments. Collected the supernatant and centrifuged at 10000g for 10 min at 4°C to pellet mitochondrial. The supernatant was retained as the cytoplasmic fraction. The nuclear pellet was resuspended in a solution of 0.34 M sucrose, 0.5 mM Mg(Ac)_2_; layered over a solution of 0.35 M sucrose, 0.5 mM Mg(Ac)_2_; and centrifuged at 1500g for 20 min at 4°C. This resulted in a cleaner nuclear pellet. Each experiment was performed in triplicate and the results were normalized to total protein.

### Measurement of lactate production

Lactate production was used as a surrogate marker of glycolysis. Briefly, A549 and A549T cells were cultured in DMEM for 24 h in 24 well plates. Levels of lactate in the culture medium were measured with a lactate Assay Kit (CMA Microdialysis AB, Kista, Sweden) according to the manufacturer’s instructions. Cell numbers at the end of experiments were calculated. The lactate production was normalized to the A549 group with the number of cells.

### [^18^F]-Fluorodeoxyglucose ([^18^F]-FDG) incorporation

FDG uptake assays were performed essentially as described [15]. The cells in 12 well-plates were washed with PBS and placed in a glucose and serum free DMEM medium, to assay FDG uptake of the cells. Then 74 kBq [^18^F] fluorodeoxyglucose (FDG) was added to the cultures and the cells were incubated at 37°C for 1h. The cells were washed 3 times with ice-cold PBS to eliminate free FDG, and then lysed by NaOH. The lysate and a standard solution were counted in a γ counter (Beckman LS6500). Cells in parallel wells were counted. The FDG incorporation was determined as the percentage of the original concentration and normalized with number of cells.

### Measurement of cell viability

Cells were seeded in 96-well plates at 5x10^3^ cells/well. After 24h, cells were treated with various drugs and incubate for 48h. Cell viability was determined using the CCK-8 assay (Dojindo) according to the manufacturer’s instructions, and normalized to the non-treatment control group.

### Statistics

Graphpad prism software was used for statistical analysis and for plotting graphs. Results are expressed as means±Standard Deviation (SD) of three replicate samples, and the significance of the differences between the means of treatment groups and controls was determined using Student’s t-test. For statistical interpretation, P<0.05 (*) is considered significant, p<0.01 (**) is considered highly significant, and P<0.001 (***) is considered very highly significant.

## Results

### The metabolic characteristics of paclitaxel-resistant and paclitaxel-nonresistant A549 cells

**Figure 1:**
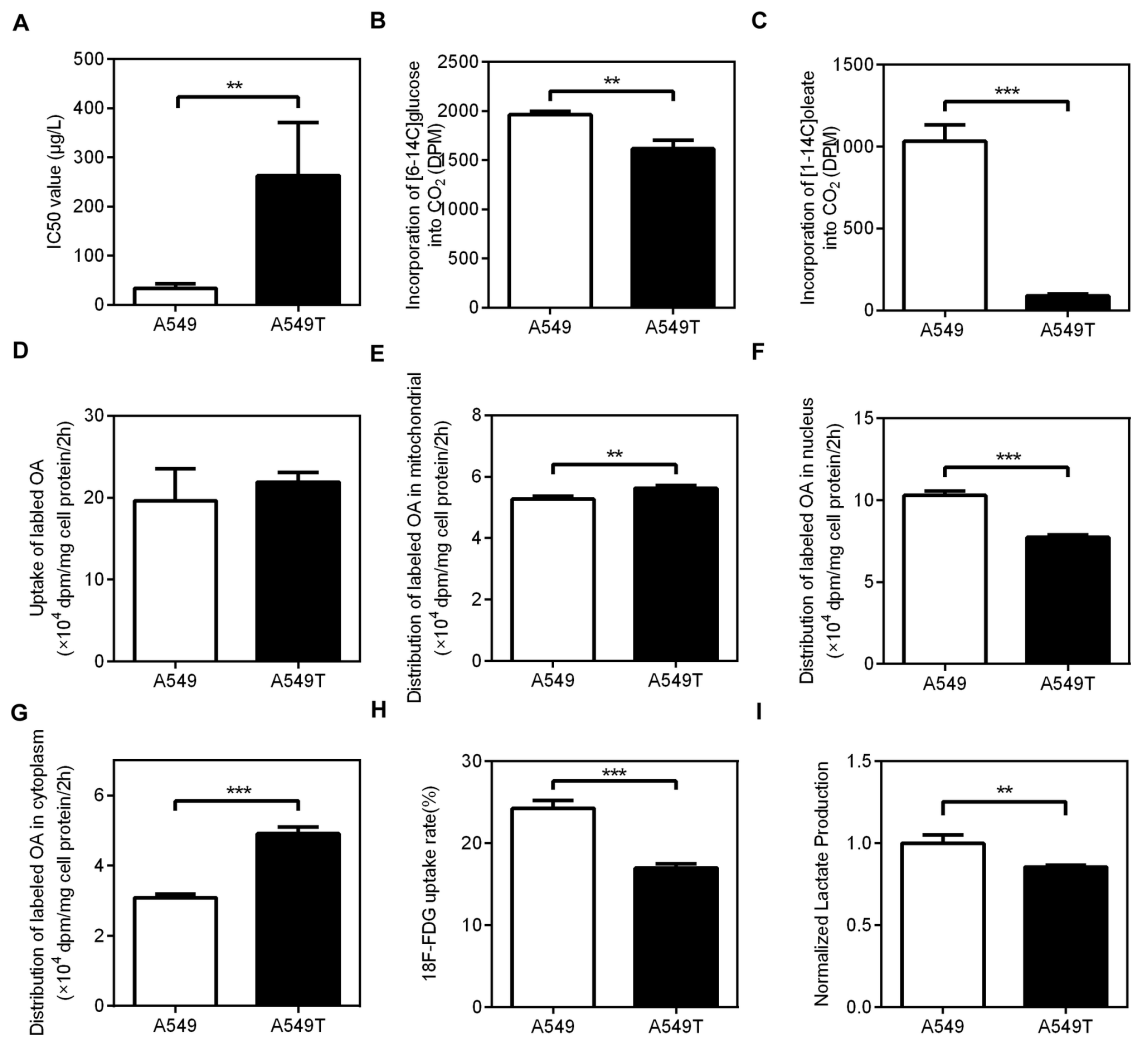
The metabolic characters of A549 and A549T cells (A) The half maximal inhibitory concentration (IC50) of paclitaxel, analyzed by CCK-8. For A549T cells, the incorporation of (B) [1-^14^C] oleate and (C) [6-^14^C] glucose into CO_2_ was 20% lower than A549, and the incorporation of [1-^14^C] oleate into CO_2_ was much lower than A549. (D) Uptake of labeled oleate (0.1µCi/mL). The distribution of labeled OA between (E) mitochondria, (F) nucleus and (G) cytoplasm. (H) ^18^F-FDG uptake and (I) lactate produced by A549T is lower than A549. Data are means ± SD (n = 3).

The paclitaxel-resistant cell line (A549T), was developed from the paclitaxel-nonresistant cell line (A549). With the relative resistance (as a resistance factor) calculated via the ratio of the half maximal inhibitory concentration (IC50)-resistant variant/IC50 of the parental cell line, the A549T cell line was 7.73-fold resistant to paclitaxel (Figure 1A). Since A549T is much less sensitive to paclitaxel than A549, a higher concentration of paclitaxel was selected for A549T in subsequent experiments.

To examine the characteristics of glycolysis and lipolysis in A549 or A549T cells, oxidation of glucose and oleate was determined by measurement of CO_2_ released from these cells (Figure 1B and 1C). Both the incorporation of [1-^14^C] oleate and [6-^14^C] glucose into CO_2_ in A549T cells was lower than that in A549 cells.

Uptake of labeled oleate (the sum of cell-associated lipids and oxidized oleate) had no significant difference between A549/A549T cells (Figure 1D). The oleate distribution in mitonchonria was similar between A549/A549T cells (Figure 1E). Incorporation of labeled oleate into nucleus of A549T showed lower than that of A549 (Figure 1F), while in cytoplasm, an increase was observed (Figure 1G).

To further study the difference between A549 and A549T on glucose transport and glycolysis^18^. F-FDG uptake and lactate production were determined. As shown in Figure 1H and 1I, both ^18^F-FDG uptake and lactate production of A549T were lower than that of A549. These data suggested that paclitaxel-resistant cells showed completely different metabolic characteristics in contrast to non-resistant lung adenocarcinoma cells.

### The combination of paclitaxel and 2DG shows less growth inhibition in resistant A549T cells than parental A549 cells

**Figure 2:**
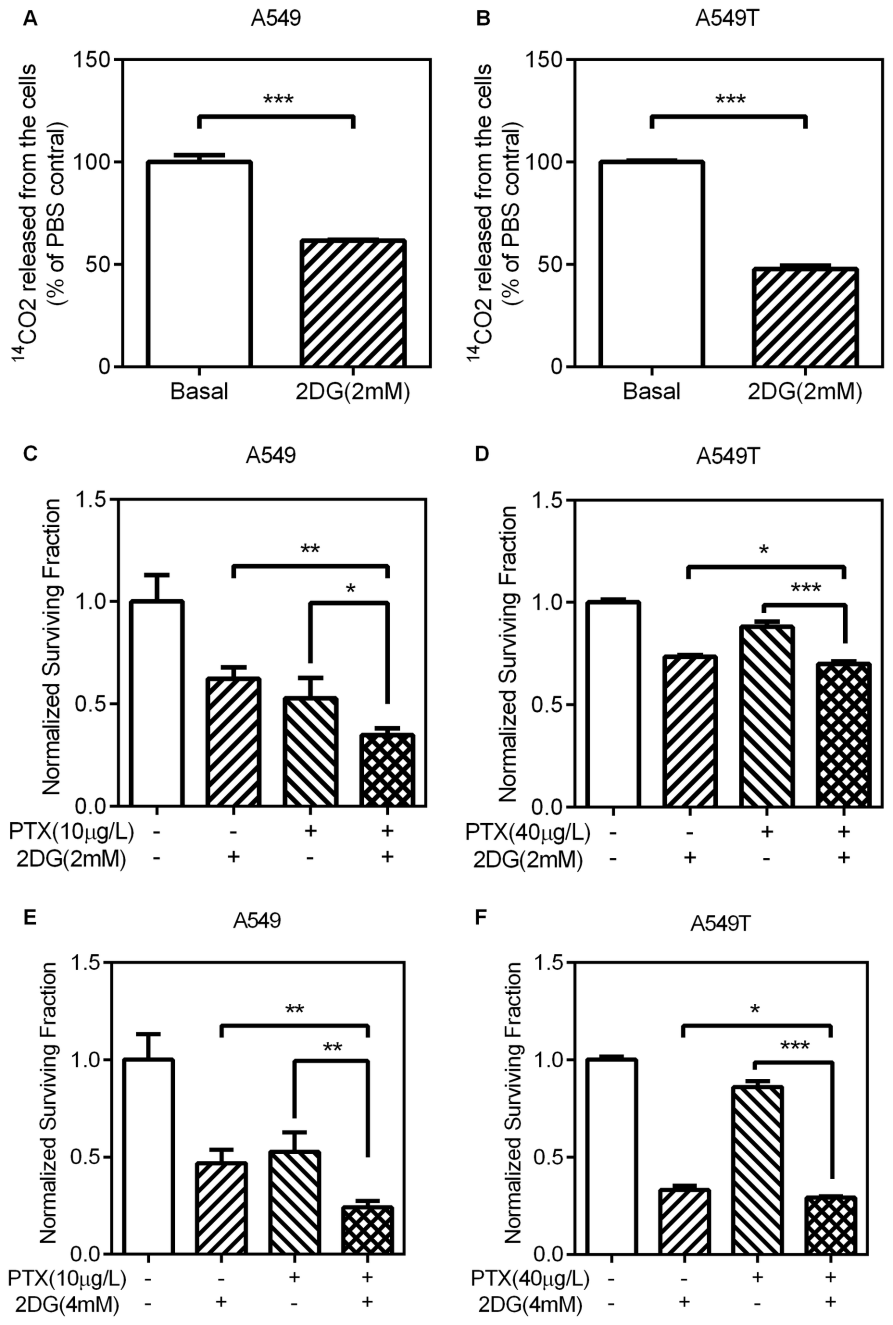
The effects of 2DG on CO_2_ production and paclitaxel sensitivity (A,B) A549 and A549T cells were treated with 2mM 2DG for 24h. The production of CO_2_ was measured as above. (C–F) A549 and A549T cells were treated with the indicated concentration of 2DG, PTX, or 2DG+PTX and incubated for 48h. Relative cell viability was measured and calculated as described in “Materials and Methods”. Treatments by 2DG+PTX have greater antiproliferative effect than each drug alone in both A549 and A549T cells. Data are means ± SD (n = 3).

2-deoxyglucose (2DG) is a glucose analog that is phosphorylated by hexokinase to 2-deoxyglucose-phosphate, which cannot be further metabolized [16,17]. 2DG has been widely used as a glycolysis inhibitor. To study the influence of 2DG on glycolysis in these lung adenocarcinoma cells, the incorporation of [6-^14^C] glucose into CO_2_ was investigated. As shown by production of ^ 14^CO_2_, the glycolysis was significantly inhibited by 2DG (2mM) in both A549 (Figure 2A) and A549T (Figure 2B) cells. To assess the effects of glycolysis inhibition on paclitaxel sensitivity, cells were incubated with 2DG, PTX or 2DG+PTX for 48h. 2DG apparently improved the sensitivity of A549 cells to paclitaxel treatment (Figure 2C and 2E), which is consistent with previous reports [18]. In contrast to paclitaxel alone, the paclitaxel-resistant A549T cells also showed further cell killing upon combination treatment of 2DG and paclitaxel (Figure 2D and 2F), but less effective compared with A549 cells. These data indicated that glycolysis inhibition was a less effective method to improve the paclitaxel sensitivity in lung adenocarcinoma cells.

### Inhibition of oxidative phosphorylation mediated by malonate had no effect on sensitivity of lung adenocarcinoma cell to paclitaxel

**Figure 3:**
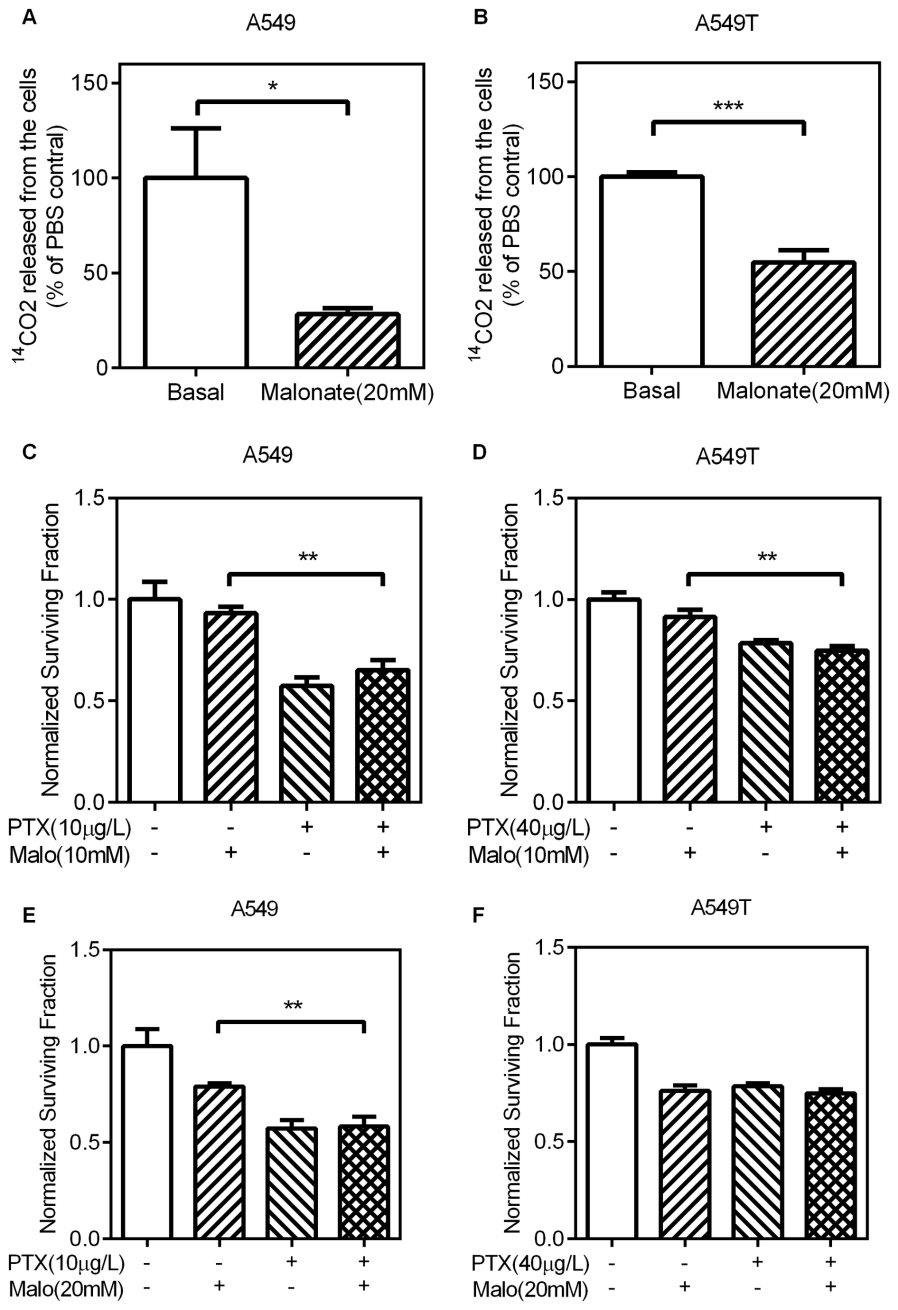
The effects of malonate on CO_2_ production and paclitaxel sensitivity (A,B) A549 and A549T cells were treated with 20mM malonate for 24h. The production of CO_2_ was measured as above. (C–F) A549 and A549T cells were treated with the indicated concentration of Malo, PTX, or Malo+PTX and incubated for 48h. Relative cell viability was measured and calculated as above. Treatments by Malo+PTX have similar antiproliferative effect compared to Malo or PTX alone in both A549 and A549T cells. Data are means ± SD (n = 3).

Malonate is a competitive inhibitor of succinate dehydrogenase, which is a key enzyme in oxidative phosphorylation [19]. Malonate binds to the active site of the enzyme without reacting, and so competes with succinate, the usual substrate of the enzyme. To confirm the influence of malonate on glucose oxidation, we examined the incorporation of [6-^14^C] glucose into CO_2_ upon treatment with malonate. In the presence of malonate, the oxidative phosphorylation was inhibited in both paclitaxel-resistant A549T (Figure 3A) and its parental A549 cells (Figure 3B). For non-resistant adenocarcinoma cells, combination of malonate and paclitaxel did not induce more cell death than paclitaxel alone (Figure 3C and 3E). Similarly, there was no difference between paclitaxel alone and combination of paclitaxel and malonate in cell killing of paclitaxel-resistant A549T cells (Figure 3D and 3F). Therefore, inhibition of oxidative phosphorylation had no effect on sensitivity of lung adenocarcinoma cells to paclitaxel.

### The fatty acid oxidation inhibitors specifically sensitized the paclitaxel-resistant of lung adenocarcinoma cells to paclitaxel

**Figure 4:**
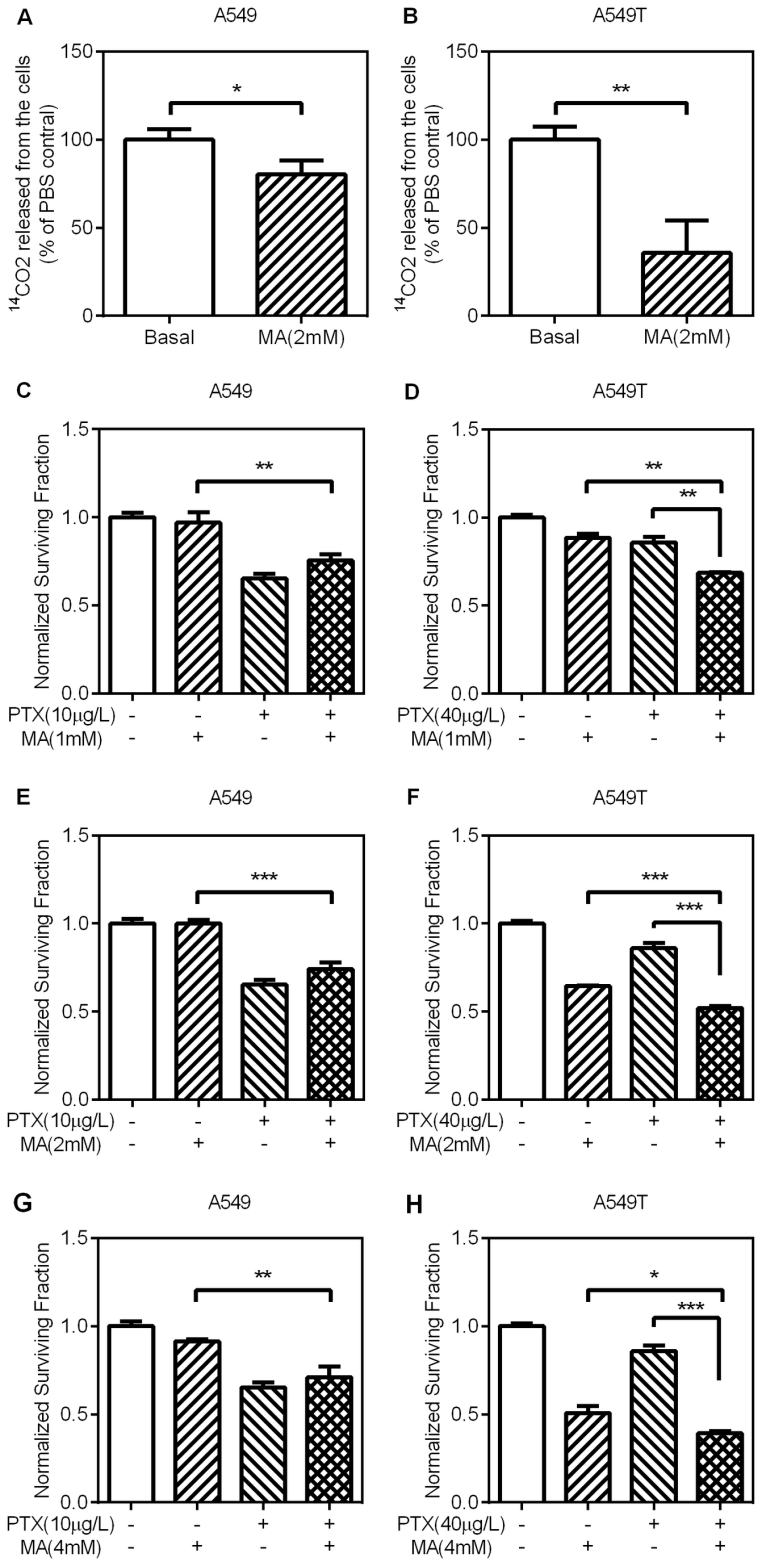
The effects of Mercaptoacetate on CO_2_ production and paclitaxel sensitivity (A,B) A549 and A549T cells were treated with 2mM MA for 24h. The production of CO_2_ was measured as above. (C–F) A549 and A549T cells were treated with the indicated concentration of MA, PTX, or MA+PTX and incubated for 48h. Relative cell viability was measured and calculated as above. Treatments by MA+PTX have similar antiproliferative effect compared to MA or PTX alone in A549 cells, but greater in A549T cells. Data are means ± SD (n = 3) Data are means ± SD (n = 3).

Mercaptoacetate (MA) inhibits the enzyme fatty acid CoA dehydrogenase, which blocks the first step in fatty acid oxidation [20,21]. To verify whether MA could alter the incorporation of [1-^14^C] oleate into CO_2_, the production of ^14^CO_2_ was measured. As shown by CO_2_ production, the fatty acid oxidation was significantly inhibited in these lung adenocarcinoma cells (Figure 4A and 4B). Next, we tested whether MA treatment had an influence on cell growth or chemo sensitivity. As shown in Figure 4C, single use of 1mM MA in nonresistant cells had no significant anti-proliferative effect. The combination of MA and paclitaxel even increased cell growth in contrast to PTX alone. However, for drug-resistant cells, 1mM MA, as a less toxic concentration, caused slightly proliferative inhibition, whereas the combination regimen apparently sensitize resistant cells to paclitaxel (Figure 4D). Consistently, 2mM or 4mM of MA could improve the sensitivity of PTX-resistant cells to paclitaxel (Figure 4E and 4G). And 2mM or 4mM of MA had no effect on sensitivity of non-resistant cells to paclitaxel (Figure 4F and 4H).

**Figure 5:**
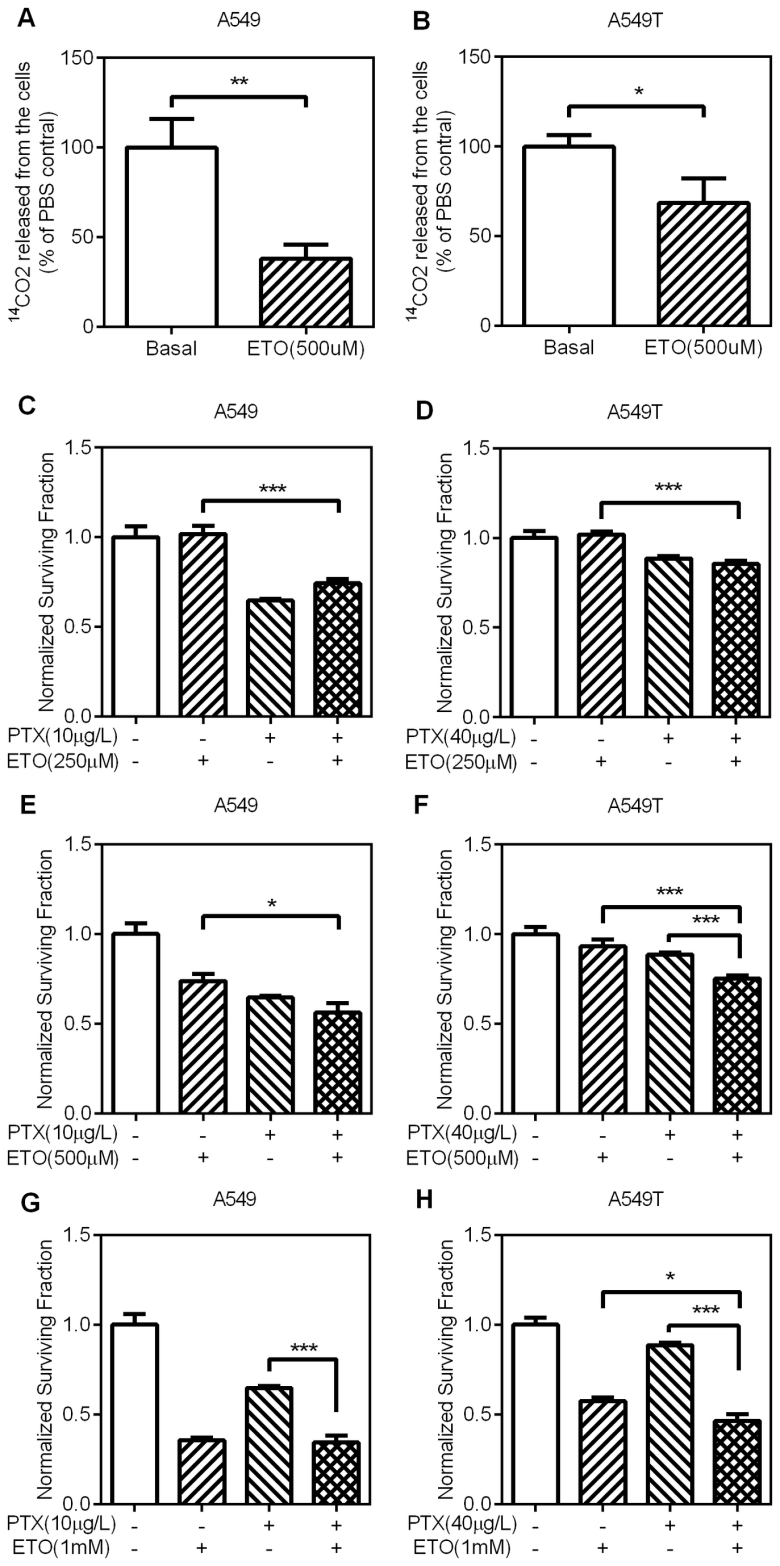
The effects of etomoxir on CO_2_ production and paclitaxel sensitivity (A,B) A549 and A549T cells were treated with 500µM ETO for 24h. The production of CO_2_ was measured as above. (C–F) A549 and A549T cells were treated with the indicated concentration of ETO, PTX, or ETO+PTX and incubated for 48h. Relative cell viability was measured and calculated as above. Treatments by ETO+PTX have similar antiproliferative effect compared to MA or PTX alone in A549 cells, but greater in A549T cells. Data are means ± SD (n = 3).

To further confirm the effect of FAO inhibitor on paclitaxel resistant of lung adenocarcinoma cells, another inhibitor was used. Etomoxir (ETO) acts by inhibiting carnitine palmitoyl transferase-1 (CPT1), the enzyme that transports long-chain fatty acids into mitochondria [22]. ETO (500µM) could apparently inhibit FAO in both A549 (Figure 5A) and A549T (Figure 5B) cells. In a lower concentration (250µM), ETO did not change the sensitivity of both resistant (Figure 5C) and non-resistant (Figure 5D) cells. For non-resistant lung adenocarcinoma cells, paclitaxel combined with 500µM (Figure 5F) or 1mM (Figure 5H) did not display any synergistic effect. However 500µM (Figure 5E) or 1mM (Figure 5G) ETO apparently improved the sensitivity of PTX-resistant cells to paclitaxel. Taken together, these results indicated that inhibition of FAO could reverse the resistance of lung adenocarcinoma cells to paclitaxel.

## Discussion

Drug resistance remains the main obstacle to cure lung cancer, which is the most common cause of cancer death worldwide [23]. Dysregulation of cellular metabolism is closely associated with major human diseases such as obesity, diabetes, and cancer [24]. More recently, there is increasing evidence that dysregulated cellular metabolism is linked to the phenomenon of chemo drug resistance [25]. Targeting dysregulated metabolism has become a promising strategy to improve the efficacy of cancer therapy. Yet it remains unknown if the metabolic transformation is a cause or a result of chemoresistance.

In this study, it was shown that the PTX-resistant lung adenocarcinoma cell A549T is different from the parental A549 cell line in glucose and fatty acid metabolism. A549T was lower in FDG uptake, lactate production and glucose oxidation. Interestingly, although the [1-^14^C] oleate uptake shown no significant difference between A549 and A549T cells, the incorporation of [1-^14^C] oleate into CO_2_ was much lower in A549T. Moreover, the distribution of labeled OA was quiet different between the two cell lines. For A549T cells, the radioactivity was lower in nucleus and higher in cytoplasm.

Compared to normal cells, tumor cell ATP production depends greatly on glycolysis and often also on fatty acid oxidation. This reprogrammed cancer metabolism, characterized by enhanced glycolysis and suppressed oxidative phosphorylation, is known as the Warburg effect. The Warburg effect is closely associated with either inherent or acquired drug resistance in cancer cells [26]. Agents that target glycolysis such as inhibitors of hexokinase 2 (HK2) and lactate dehydrogenase-A have shown promising efficacy in the conversion of drug resistance in several in vitro models. It was reported that inhibition of glycolysis by 3-bromopyruvate could overcome drug resistance in malignant cells through the inactivation of ATP-binding cassette transporters, ATP-dependent efflux pumps [27]. For 2DG, an inhibitor of HK2, was shown that could overcoming trastuzumab resistance in breast cancer [28]. The combination of 2DG and paclitaxel could enhance breast cancer cell killing [29]. In this study, we have demonstrated that paclitaxel combined with 2DG shown potentiated antiproliferative effect in non-resistant cell line, but for drug resistant cells, the potentiation effects were much weaker. Thus we may conclude that, compared with the sensitive cells, the combination of paclitaxel and glycolysis inhibitors may yield less therapeutic benefits for drug resistant cells.

Malonate, an inhibitor of succinate dehydrogenase and mitochondrial ETC complex II, could effectively inhibit oxidative phosphorylation (OXPHOS). The inhibition of oxidative phosphorylation or ETC, in particular, has been shown could inhibit cell death induced by chemo-drugs [30]. Here, we found that though malonate could inhibit both paclitaxel-resistant and nonresistant cells by single use, but the combination of paclitaxel and malonate shown no synergistical effect, which indicate that the inhibition of OXPHOS may induce paclitaxel resistant in lung adenocarcinoma cell line.

Pharmacologic inhibition of FAO with etomoxir, an blocker of Carnitine palmitoyl transferase 1 (CPT), has reduced myeloma cells proliferation by 40–70% in a previous report [31]. Fatty acid oxidation has been shown linked to chemoresistance [6]. Additionally, it has been reported that inhibition of fatty acid oxidation with etomoxir or ranolazine sensitizes leukemia cells to apoptosis induction by ABT-737 [32]. Thus the inhibition of FAO could potentially become a novel therapeutic approach to drug resistance. Mercaptoacetate and etomoxir as fatty acids oxidation inhibitor, were used in this study to determine whether FAO inhibition could reduce the viability of drug resistant cells more effectively, or whether it could potentiate chemo-drugs on cell killing. We found that MA could inhibit paclitaxel-resistant cells more effective than nonresistant cells. Even though single use of etomoxir shown similar antiproliferative effects on A549 and A549T cells, the combination of paclitaxel with etomoxir shown potentiated effect on inhibition of A549T cell viability. Finally, we conclude that the inhibition of FAO could potentially be an effective therapeutic approach to chemo-resistant lung adenocarcinoma.

In summary, drug resistant lung adenocarcinoma cells showed reduced glucose and fatty acid oxidation, whereas the inhibition of glucose and lipolysis metabolism did not always induce drug resistance. Though the inhibition of oxidative phosphorylation may slightly induce lung adenocarcinoma cells less sensitive to paclitaxel, the combination of glycolysis/lipolysis inhibitors and paclitaxel could synergistically inhibit the proliferation of drug resistant cells. Thus we may conclude that the dysregulated metabolism is an accompanied phenomenon rather than a contributor of drug resistance. On the other hand, paclitaxel combined with inhibitors of glycolysis and lipolysis metabolism could synergistically inhibit drug-resistant lung adenocarcinoma cell proliferation. Targeting different energy metabolic pathways showed quite different effects on cell proliferation. For drug resistant cells, in particular, inhibition of lipolysis enzymes could be a promising strategy to improve the efficacy of cancer therapy.
